# Breaking the Ice and Forging Links: The Importance of Socializing in Research

**DOI:** 10.1371/journal.pcbi.1003355

**Published:** 2013-11-21

**Authors:** Miranda Stobbe, Tarun Mishra, Geoff Macintyre

**Affiliations:** 1 Bioinformatics Laboratory, Department of Clinical Epidemiology, Biostatistics and Bioinformatics, Academic Medical Center, University of Amsterdam, Amsterdam, The Netherlands; 2 Netherlands Bioinformatics Centre, Nijmegen, The Netherlands; 3 Bioinformatics Division, School of Bio Sciences and Technology, VIT University, Tamil Nadu, India; 4 NICTA Victoria Research Laboratory, Department of Electrical and Electronic Engineering, The University of Melbourne, Victoria, Australia; 5 Department of Computing and Information Systems, The University of Melbourne, Victoria, Australia; Princeton University, United States of America

## Abstract

When meeting someone for the first time—whether another PhD student, or the Founding Editor-in-chief of *PLOS Computational Biolog*y—nothing breaks the ice like eating pancakes or having drinks together. A social atmosphere provides a relaxed, informal environment where people can connect, share ideas, and form collaborations. Being able to build a network and thrive in a social environment is crucial to a successful scientific career. This article highlights the importance of bringing people together who speak the same scientific language in an informal setting. Using examples of events held by Regional Student Groups of the ISCB's Student Council, this article shows that socializing is much more than simply sharing a drink.

## Socializing in Science

Clear communication of research ideas and results is vital in order to become a successful scientist. Although conference presentations are an efficient way to share results, the one-way nature of this type of communication does not lend itself to collaboration. Setting up collaborations is often better achieved in an informal social setting, which provides an intimate environment with time to discuss things in detail. A social setting is where new colleagues are found, collaborations are established, and existing connections are strengthened.

Social settings make networking easier by reducing boundaries to communication. Typically, after a keynote presentation at a conference there are relatively few questions from young scientists. This is due to a communication barrier—the junior scientist may be intimidated by the achievements of the senior scientist, or may be afraid to ask a question that might be deemed “stupid.” A social setting can remove the communication barrier, as the threshold for asking a question in front of a highly critical and expert audience is far higher than asking a question when sharing a drink together.

More often than not, in fact, experts enjoy discussing their science with young scientists and appreciate that a fresh point of view can provide stimulating discussion. Organized social events can therefore be helpful, especially for those who don't characteristically initiate contact with strangers. An event or activity that provides the opportunity for introductions can ease any awkwardness and improve networking.

## The Importance of Networking

In a multidisciplinary research field like computational biology, it is crucial to have an environment where scientists can connect and communicate directly. PhD students and postdocs will often work in a research group that consists of scientists with diverse educational backgrounds. The supervisor of a PhD student may not have a background in computational biology. Therefore, it is necessary to create a supporting network that can be called on to share experiences. Moreover, a solid network becomes a crucial element for future collaborations and taking the next steps in one's career.

As junior scientists are likely to start with a limited network in their scientific field, they should look at social events as not only a way to unwind but also a tool to further a career. To this end, it is important for organizers of formal events to incorporate opportunities for socializing in their program, which has the added benefit of ensuring more interaction between event participants. Furthermore, students should be encouraged by their PIs to go to social events, as being able to succeed in a social setting is critical to becoming a well-rounded scientist.

A social event can be as much about making friends as it is about sharing research—you are more likely to remember someone based on a shared interest like playing the guitar than his/her particular research topic. Ultimately though, the connection made may lead back to better science and increased opportunities for collaboration.

## How to Break the Ice

The phrase “practice makes perfect” applies to many situations, and socializing is no exception, although the phrase “all things in moderation” may also apply, depending on the individual. Nevertheless, the greater the exposure to socializing opportunities, the better one's communication skills get, which is a vital skill in science.

Across the world, Regional Student Groups (RSGs) have organized social events in all shapes and sizes, taking into account what will work best in the specific country. Stand-alone meetings in a pub or at a university have proven to be the event of choice in some countries. In other countries, especially the larger ones, linking a social event to a more formal event like a (national) conference is the preferred option. These types of events have even been extended to international conferences bringing together people from many countries. In each case, however, the common theme has been to provide a relaxed atmosphere. These RSG-organized social events provide great examples of events well-suited for scientific networking.

A popular concept for many of the events is a combination of a scientific or soft-skill-related discussion with food and drinks during and/or afterward. RSG Australia organizes social events that consist of meeting for food and drinks and talking all things bioinformatics in an informal atmosphere. Senior researchers are encouraged to attend, to help stimulate conversation. As many of the students have multiple supervisors in disparate disciplines (computer science, molecular biology, statistics—to name a few), a conversational environment filled with people who speak the same language is welcomed. The event is always held at a location not affiliated with any particular university or institution to encourage diverse participation. Similar types of social events have been organized by RSG France (scientific breakfast and pub meetings) and RSG Netherlands (BioCafé), in which the social event provides participants with both a possibility to extend their network and another means of acquiring knowledge.

RSG India has adopted a different approach and organized a series of journal clubs. In contrast to traditional science clubs at Indian universities, RSG India's journal clubs are much more relaxed and students are encouraged to participate irrespective of their level of understanding of the paper. This is particularly good for junior researchers, for whom reading an article can be as hard as the proverbial rocket science. It also allows more senior students or young investigators to hone their mentorship skills. RSG India is currently running two such journal clubs, and the turnout is always impressive. Club meetings are held once a fortnight with no registration fee. They are immensely popular in India for their unique concept and approach.

Another highly successful concept is “scientific speed dating,” as first organized by RSG Netherlands the evening before the Benelux Bioinformatics Conference in 2008, and since adopted at many Student Council meetings. This has proved to be an excellent way to break the ice between participants. These events are like regular speed dating—participants are randomly paired and get three minutes to talk to each other—but with one twist: participants focus on their research topic, rather than their sex appeal. After each three-minute conversation, the participants switch to the next “partner.” In this way, they get an idea of the (scientific) interests of each of the other participants. Based on this, participants can decide with whom it might be interesting to have a more lengthy discussion. An added bonus is that the participants practice describing their research in less than 3 minutes. This type of social event is easy to organize and ensures more interaction during formal events following these brief “dates.”

RSG social events such as these have been shown to be highly successful in fulfilling their intended purpose: breaking the ice and forging links.

## Tips and Tricks for Organizing a Social Event

For these events to be useful, it is imperative to run them regularly and effectively. Organizing social events is itself a very useful skill, and not without challenges. For example, during initial sessions the excitement about social events is high, but once the novelty wears off, turnout declines. It can become tedious to keep people interested. Interest can wane, even in free events, which can be discouraging for organizers. Not all people who register for an event show up, especially if the event is free of charge, which may result in a much smaller audience than anticipated. Consequently, the organizers might end up with a disappointed speaker or paying for meals for people who were not actually there.

Events that require an investment need consistent funding to keep the show running. Unfortunately, there are often not enough funding sources to sponsor such events. Club membership is one way to overcome this; however, this may not appeal to students, who have limited sources of income. Even aside from income, having to apply (and pay) for a membership may simply take too much effort for many students. Nevertheless, a few enthusiastic heads can come up with great, cheap ideas to create an environment for like-minded individuals to connect.

To make sure a social event is a success, we provide a set of tips and tricks drawing on the experience of many RSGs. Firstly, the key to deciding on the right format for a social event is to keep in mind the cultural barriers that may exist in a particular country. The pub events organized by RSG France and Netherlands would not work in countries such as India, where social drinking is not (yet) accepted among men and women equally. Related to this, the impact of a generation gap between junior and senior scientists will also differ from country to country. Secondly, social events are easy to run as part of a more formal event such an international conference. People are generally excited about their favorite conference and have it marked on their calendar every year. By attaching a social event to a conference, it gives the event broader visibility and participation. People will also associate the social event with the conference, therefore making a lasting impression. The event can be organized during a break in workshops and guest lectures, to make the agenda less tiring. Short quizzes and games can be used to break the monotony. Thirdly, taking a cue from the journal clubs in India and the BioCafé in the Netherlands, stand-alone social events can also do well. The format of the event should be flexible yet have an identity of its own. However, keep in mind that it may be challenging to keep participants turning up every time. Young researchers are one of the best pools of potential attendees to tap into. Their curiosity levels and diverse research backgrounds make the event bustling and lively, and free food will make sure their salary-deprived bellies will welcome the event.

Social events are knowledge-sharing, fun, and business packed in one. Traditionally, networking in science has been through conferences or similar formal events. However, scientists need to be able to convey the relevance of their findings to a wide audience in various platforms. You don't always need a 20-minute presentation to get an idea across—a napkin at a social event may suffice. Young researchers need to be encouraged to join in social events and not solely rely on formal scientific events. Communication is key in any scientific discipline and highly interactive events will encourage this.

**About the Authors**

**Geoff Macintyre:** ISCB Student Council, chair (2011–2012), RSG Australia, co-founder and president (2009–2010)

**Miranda Stobbe:** RSG Netherlands secretary (2008–2012)

**Tarun Mishra:** RSG India president (2010–2012)

